# Supplementary material for: Topological magneto-optical effects and their quantization in noncoplanar antiferromagnets
Source: Nat Commun. 2020 Jan 8;11:118. doi: 10.1038/s41467-019-13968-8 (PMC6949225; doi:10.1038/s41467-019-13968-8)
Supplement: Supplementary file 1 — Supplementary Information [file 41467_2019_13968_MOESM1_ESM.pdf]

Topological magneto-optical effects and their quantization in noncoplanar antiferromagnets

Feng et al.

Supplementary Figures

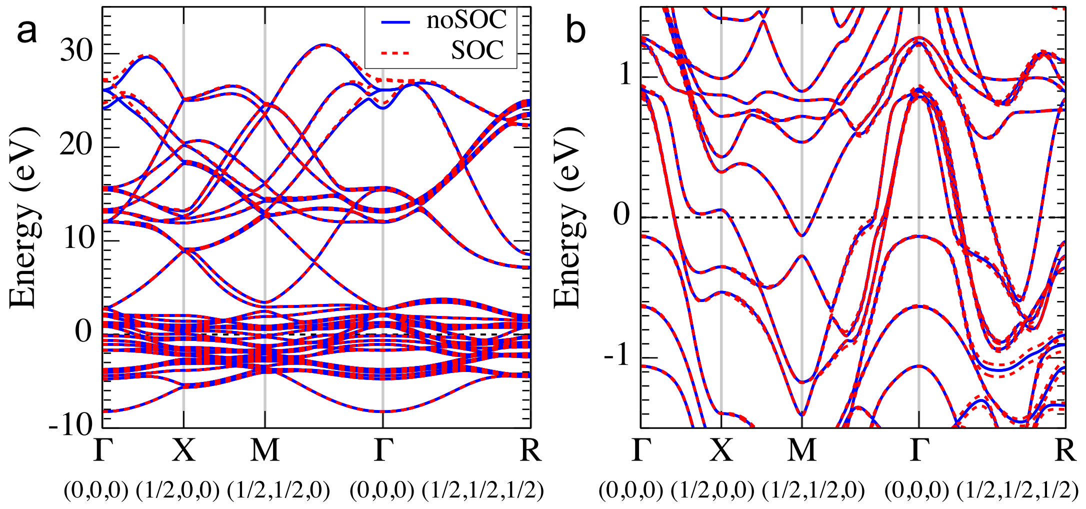

Supplementary Figure 1. Band structures of γ-$Fe_{0.5}Mn_{0.5}$. **a** Band structures of γ-$Fe_{0.5}Mn_{0.5}$ without and with spin-orbit coupling under the strain δ=0.95. **b** The enlarged band structures within the energy range of [-1.5~1.5] eV. The bands are twofold spin degenerate unless the spin-orbit coupling is switched on.

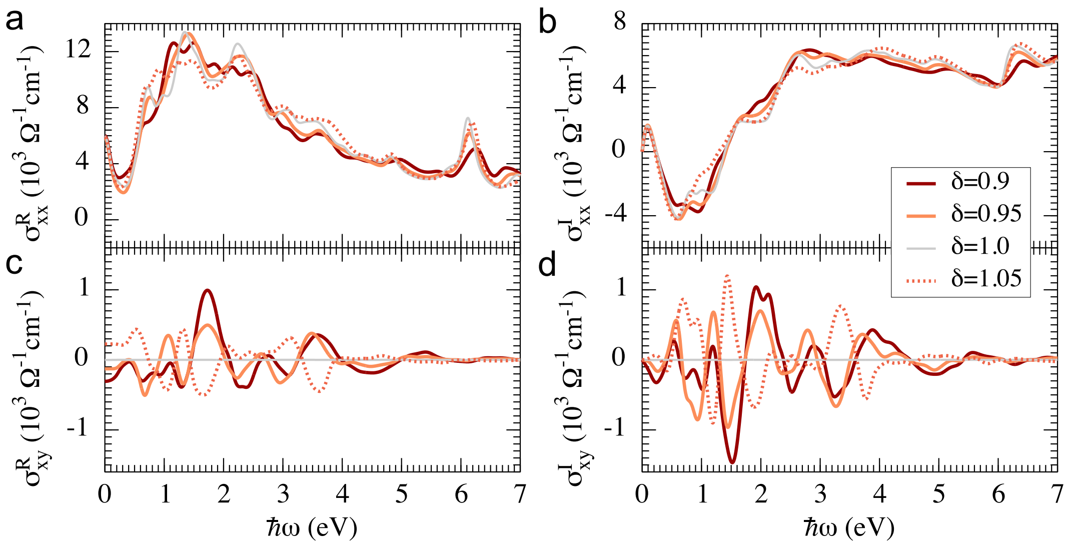

Supplementary Figure 2. The optical conductivities of γ-$Fe_{0.5}Mn_{0.5}$ under various strains. **a,b** The diagonal elements have similar profiles regardless of strains. The Drude term has been added to the diagonal elements. **c,d** The off-diagonal elements (i.e., the magneto-optical conductivity) arise only after the strains are applied. In particular, the magneto-optical conductivity can be effectively tuned by the strain, that is, the magnitude of $\sigma_{xy}(\omega)$ is proportional to $|\delta - 1|$ and the sign of $\sigma_{xy}(\omega)$ changes along with $sgn(\delta - 1)$. The spin-orbit coupling is not included in the calculations.

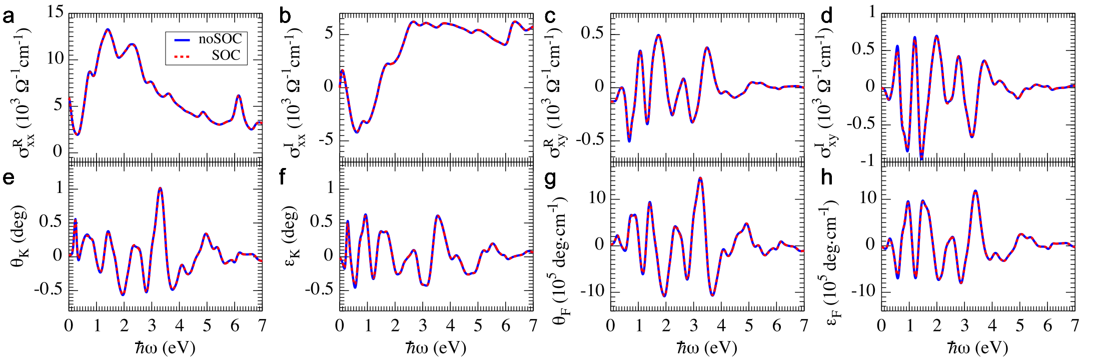

Supplementary Figure 3. The optical conductivity and magnet-optical spectrum of γ-$Fe_{0.5}Mn_{0.5}$. The optical conductivity **a-d** as well as the magneto-optical Kerr **e,f** and Faraday **g,h** spectra without and with spin-orbit coupling. The Drude term is included and the strain δ=0.95 is applied along the [111] direction. Spin-orbit coupling does not influence the magneto-optical effects in γ-Fe_xMn_{1-x}, demonstrating the topological origin of magneto-optical effects.

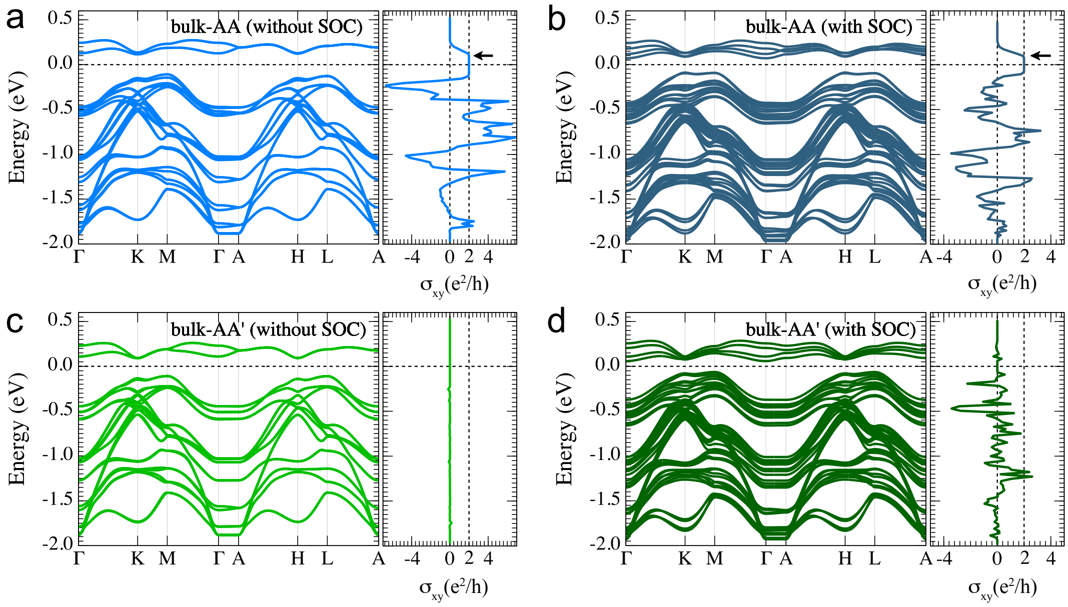

Supplementary Figure 4. The band structures and anomalous Hall conductivities of $K_{0.5}RhO_2$. **a,b** The band structures and anomalous Hall conductivities of the AA stacked $K_{0.5}RhO_2$ without and with spin-orbit coupling. **c,d** The same as **a,b** but for the AA' stacked $K_{0.5}RhO_2$. The bands are twofold spin degenerate without spin-orbit coupling. The AA stacked $K_{0.5}RhO_2$ is a quantum topological Hall insulator with the Chern number C=2. On the other hand, the AA' stacked $K_{0.5}RhO_2$ is a normal insulator with the Chern number C=0. The anomalous Hall conductivities of $K_{0.5}RhO_2$ are scaled by the length of the crystallographic z axis such that the unit is e^2/h in accord with that of two-dimensional systems.

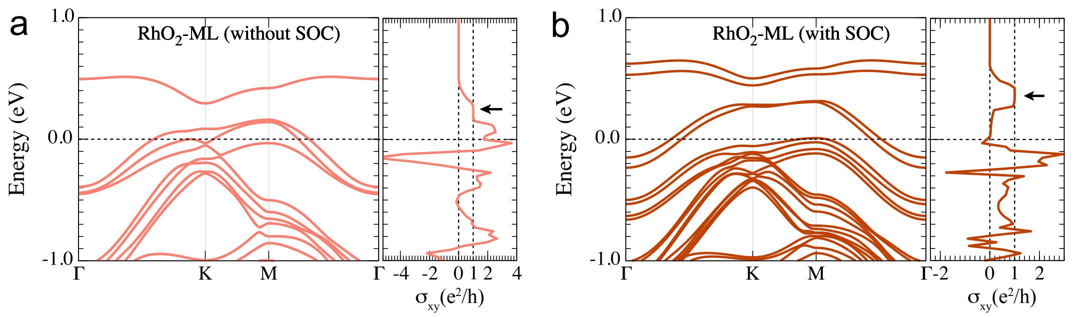

Supplementary Figure 5. The band structures and anomalous Hall conductivities of RhO_2 monolayer. **a** The band structures and anomalous Hall conductivities of RhO_2 monolayer without spin-orbit coupling. **b** The same as **a** but with spin-orbit coupling. The bands are twofold spin degenerate without spin-orbit coupling. RhO_2 monolayer is metallic intrinsically. However, it turns out to be a quantum topological Hall insulator with the Chern number C=1, if the Fermi energy is moved into the band gap above, e.g., by electron doping.

Supplementary Tables

	AH family (driven by an electric field)	MO family (driven by an optical field)
Spin-orbit coupling + *band spin splitting*	AH effect[a]	MO effect[e]
	QAH effect[b]	QMO effect[f]
Scalar spin chirality	TH effect[c]	TMO effect[*]
	QTH effect[d]	QTMO effect[*]

Supplementary Table 1. The family members of the anomalous Hall (AH) and magneto-optical (MO) effects as well as their distinctly different origins. QAH: quantum anomalous Hall; QMO: quantum magneto-optical; TH: topological Hall; TMO: topological magnet-optical; QTH: quantum topological Hall; QTMO: quantum topological magneto-optical.

[a] Supplementary Reference 1 and references therein;
[b] Supplementary Reference 2 and references therein;
[c] Supplementary References 3 and 4 and references therein;
[d] Supplementary Reference 5 and references therein;
[e] Supplementary References 6-9 and references therein;
[f] Supplementary References 10-12 (Theory) and 13-17 (Experiment);
[*] The present work.

Supplementary References,

1. Nagaosa, N., Sinova, J., Onoda, S., MacDonald, A. H. & Ong, N. P. Anomalous Hall effect. *Rev. Mod. Phys.* **82**, 1539 (2010).
2. Liu, C.-X., Zhang, S.-C. & Qi, X.-L. The Quantum Anomalous Hall Effect: Theory and Experiment. *Annu. Rev. Condens. Matter Phys.* **7**, 301 (2016).

3. Shindou, R. & Nagaosa, N. Orbital ferromagnetism and anomalous Hall effect in antiferromagnets on the distorted fcc lattice. *Phys. Rev. Lett.* **87**, 116801 (2001).
4. Ohgushi, K., Murakami, S. & Nagaosa, N. Spin anisotropy and quantum Hall effect in the kagomé lattice: Chiral spin state based on a ferromagnet. *Phys. Rev. B* **62**, R6065 (2000).
5. Zhou, J., Liang, Q.-F., Weng, H., Chen, Y. B., Yao, S.-H., Chen, Y.-F., Dong, J. & Guo, G.-Y. Predicted Quantum Topological Hall Effect and Noncoplanar Antiferromagnetism in $K_{0.5}RhO_2$. *Phys. Rev. Lett.* **116**, 256601 (2016).
6. Reim, W. and Schoenes, J. Handbook of Magnetic Materials, edited by Wohlfarth, E. P. and Buschow, K. H. J. Vol. 5, Chap. 2. *Elsevier, New York* (1990).
7. Ebert, H. Magneto-optical effects in transition metal systems. *Rep. Prog. Phys.* **59**, 1665 (1996).
8. Antonov, V., Harmon, B. & Yaresko, A. Electronic Structure and Magneto-Optical Properties of Solids. Chap. 1.4. *Kluwer Academic Publishers, Dordrecht* (2004).
9. Kuch, W., Schäfer, R., Fischer, P. & Hillebrecht, F. U. Magnetic Microscopy of Layered Structures. Chap. 2. *Springer-Verlag Berlin Heidelberg* (2015).
10. Volkov, V. A. & Mikhailov, S. A. Quantization of the Faraday effect in systems with a quantum Hall effect. *JETP Lett.* **41**, 476–478 (1985).
11. Tse, W.-K. & MacDonald, A. H. Giant magneto-optical Kerr effect and universal Faraday effect in thin-film topological insulators. *Phys. Rev. Lett.* **105**, 057401 (2010).
12. Maciejko, J., Qi, X.-L., Drew, H. D. & Zhang, S.-C. Topological quantization in units of the fine structure constant. *Phys. Rev. Lett.* **105**, 166803 (2010).
13. Wu, L., Salehi, M., Koirala, N., Moon, J., Oh, S. & Armitage, N. P. Quantized Faraday and Kerr rotation and axion electrodynamics of a 3D topological insulator. *Science* **354**, 1124–1127 (2016).
14. Dziom, V. et al. Observation of the universal magnetoelectric effect in a 3D topological insulator. *Nat. Commun.* **8**, 15197 (2017).
15. Okada, K. N. et al. Terahertz spectroscopy on Faraday and Kerr rotations in a quantum anomalous Hall state. *Nat. Commun.* **7**, 12245 (2016).
16. Shuvaev, A., Dziom, V., Kvon, Z. D., Mikhailov, N. N. & Pimenov, A. Universal Faraday rotation in HgTe wells with critical thickness. *Phys. Rev. Lett.* **117**, 117401 (2016).
17. Mondal, M. et al. Electric field modulated topological magnetoelectric effect in Bi_2Se_3. *Phys. Rev. B* **98**, 121106 (2018).
